# Reassortment in infectious bursal disease virus (IBDV) and related birnaviruses

**DOI:** 10.1128/jvi.01724-25

**Published:** 2026-06-24

**Authors:** Andrew J. Broadbent

**Affiliations:** 1 Department of Animal and Avian Sciences, University of Maryland 1068 , College Park, Maryland, USA; Universiteit Gent, Merelbeke, Belgium

**Keywords:** birnavirus, infectious bursal disease virus, IBDV, infectious pancreatic necrosis virus, IPNV, reassortment

## Abstract

The *Birnaviridae* family consists of non-enveloped viruses with a single capsid that have a double-stranded RNA genome divided into two segments. Because they infect birds, fish, rotifers, marine mollusks, and insects, these viruses are of major economic significance to the global poultry and aquaculture industries. In the last decade, reassortment events have led to the emergence and spread of new strains of infectious bursal disease virus (IBDV) in poultry that could complicate control efforts. This review summarizes the literature regarding the molecular epidemiology and pathogenesis of reassortant birnaviruses, discusses potential reservoirs of gene segments, and highlights gaps in our knowledge of the molecular basis underpinning the phenomenon.

## INTRODUCTION

**Fig 1:**
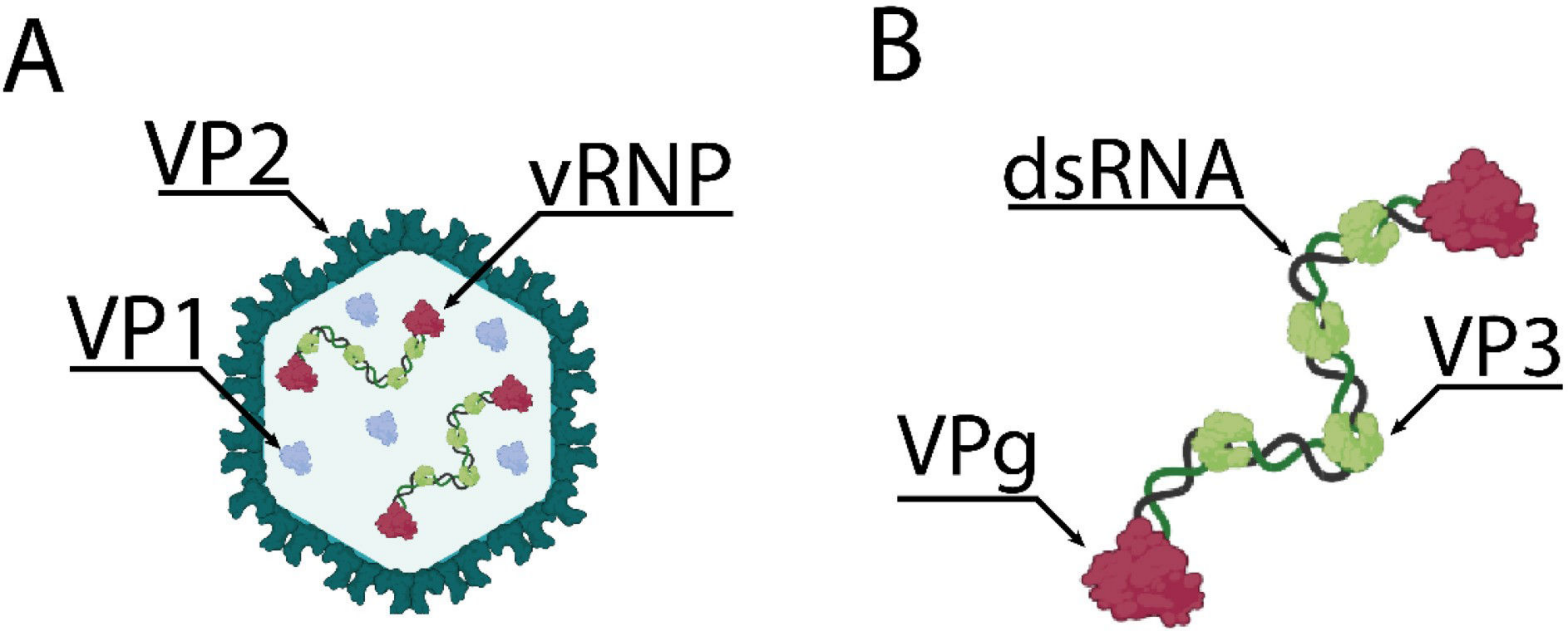
Simplified cartoon of the IBDV virion and vRNP. (**A**) The IBDV virion is comprised of an icosahedral T = 13 capsid of VP2, containing free VP1 and genomic vRNPs. (**B**) The structure of an IBDV genomic vRNP, comprising dsRNA associated with VP3 and VP1 linked to the 5′ end of each genomic strand to form a viral protein genome-linked (VPg) cap (reproduced from reference 5, previously published under a CC BY license).

The *Birnaviridae* family is comprised of viruses with genomes of double-stranded (ds) RNA, divided into two segments, A and B, packaged within a single-layered capsid with T = 13 icosahedral symmetry (Fig. 1). The family includes seven genera (1), four of which infect aquatic organisms. The *Aquabirnavirus* genus infects finfish and includes infectious pancreatic necrosis virus (IPNV) of salmon and trout, which is of major economic importance to aquaculture; the *Blosnavirus* genus infects seabass/barramundi and Blotched snakehead fish; the *Telnavirus* genus infects aquatic bivalve mollusks and includes Tellina virus 1 (TV1) of oysters; and the *Ronavirus* genus infects rotifers. The following two genera infect insects: *Dronavirus,* which includes Drosophila B birnavirus, and *Entomobirnavirus,* which includes Drosophila X virus (DXV), mosquito X virus (MoXV), Culex Y virus (CuYV), culicine-associated Z virus (CAZV), Espírito Santo virus (ESV)*,* and Eridge virus (EV). Finally, the *Avibirnavirus* genus infects birds and includes infectious bursal disease virus (IBDV), which is of major economic importance to the poultry industry. Aside from these genera, a number of divergent birnaviruses remain unassigned to any genus within the family, including Wēnlĭng jack mackerels birnavirus and Wēnlĭng Japanese topeshark birnavirus of finfish, Húbĕi birnavirus of nematodes, and chicken proventricular necrosis virus (CPNV) of birds (1). Furthermore, viral metagenomics analyses have revealed the sequences of a porcine birnavirus (2), a porcupine birnavirus (3), and a bat fecal-associated birna-like virus (4), suggesting that the *Birnaviridae* can also infect mammals.

**Fig 2:**
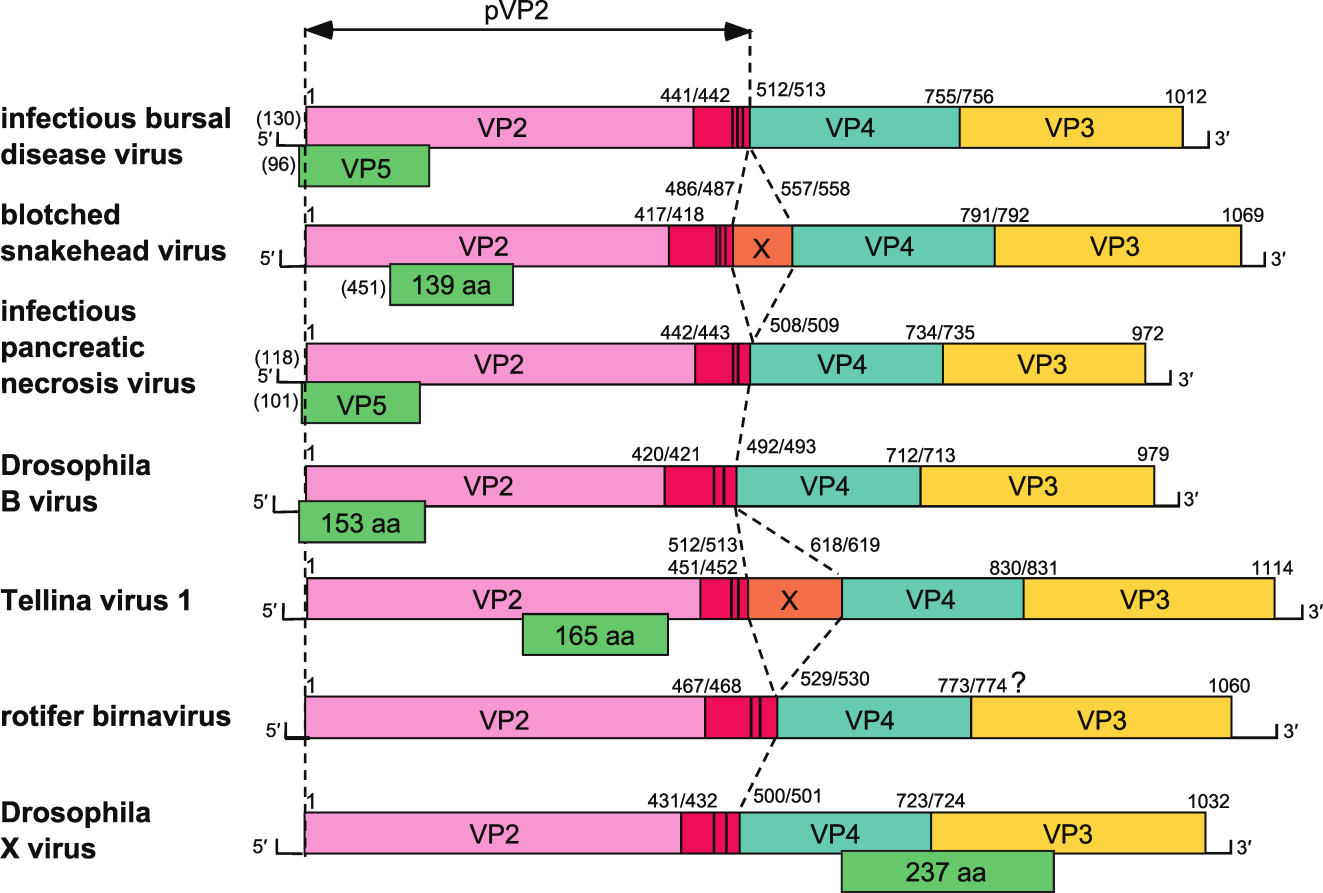
Schematic of the gene arrangement in the coding strand of genome segment A of representative birnaviruses. Polyprotein cleavage sites are indicated by a vertical bar and identified by amino acid position. Related ORFs are color-coded, with red indicating the peptides derived from processing of preVP2 and green indicating the small additional ORFs. Parentheses at the 5′ ends indicate the length of the 5′ non-coding region where known (reproduced from reference (1), previously published under a CC BY license).

Segment B encodes viral protein (VP)1, which is the RNA-dependent RNA polymerase, and segment A encodes a polyprotein, which is co-translationally cleaved by the embedded VP4 protease into its constitutive parts, releasing preVP2 (pVP2), VP4, and multifunctional protein VP3. The pVP2 is then further proteolytically cleaved at the C-terminus as the protein matures into the capsid protein VP2. An additional protein, sometimes known as VP5, is encoded by an alternative open reading frame (ORF), the location of which varies depending on the birnavirus (Fig. 2). Additionally, the blotched snakehead virus and TV1 encode polypeptide X (Fig. 2), a nonstructural protein, the function of which remains poorly understood (1).

Given the segmented nature of the genome, reassortment in any genus is a possibility; however, this has only been extensively observed within the *Avibirnavirinae*. For example, PubMed searches conducted on 9 February 2026 revealed 88 results for “IBDV reassortment,” whereas only six results for “IPNV reassortment.” Searches for other birnaviruses did not reveal any results regarding reassortment. This review summarizes the literature regarding the molecular epidemiology and pathogenesis of reassortant birnavirus strains, discusses potential reservoirs of gene segments, and highlights gaps in our knowledge of the molecular basis underpinning the phenomenon.

## IBDV REASSORTMENT

### Introduction to IBDV

IBDV causes an immunosuppressive disease in chickens due to the virus preferentially infecting and lysing B lymphocytes, which are typically found in high densities in the bursa of Fabricius (BF). Infected birds are more susceptible to secondary infections, exacerbating the impact on the welfare of the birds and on the productivity of the poultry industry. IBDV strains were first reported in Gumboro, Delaware, USA, in the 1960s, and two serotypes were subsequently identified (6–8). Serotype 2 IBDV strains are infectious to chickens but do not typically cause clinical signs or bursal pathology (9), although one study reported a reduced size of the BF in chickens inoculated at 1 day of age (10). In contrast, serotype 1 strains are of significant consequence to the global poultry industry. Initially, serotype 1 strains were classified based on pathotype, with so-called classical strains being associated with depression, ruffled feathers, and a cloacal discharge, with mortality ranging from approximately 10% to 50%, depending on the age and breed of bird (6). Disease was controlled by vaccination, initially using inactivated oil emulsion vaccines based on antigen derived from infected bursae to vaccinate layer hens, thereby providing maternal immunity to chicks. Live-attenuated vaccines, generated by serial passage of field strains in cell-culture or embryonated eggs, were developed later for vaccination of chicks after maternal immunity had waned sufficiently. IBDV strains evolved by antigenic drift in the VP2 capsid gene, leading to the accumulation of amino acid substitutions in an area known as the hypervariable region (HVR), which is thought to be responsible for receptor binding, and the region most targeted by neutralizing antibodies and therefore under the most immune selection pressure. This led to the emergence of antigenically variant strains in the 1980s in the US, which presented asymptotically but nevertheless led to lasting immunosuppression and susceptibility to secondary infections (6–8). In addition, very virulent (vv) strains also emerged in the 1980s in Europe and spread across the globe, responsible for more severe clinical disease and high mortality that reached 100% depending on the breed and age of the flock. The vv strains presented with sudden death and caused a severe lymphoid depletion in the BF (6–8). Moreover, the vv strains were able to break through maternally derived immunity, requiring the introduction of “hotter” vaccines that could be used earlier in the face of higher levels of maternal immunity.

By the 2000s, the taxonomy of IBDV had been revised into genogroups based on the sequence of the VP2 capsid HVR. Initially, seven genogroups were described by Michel & Jackwood (G1–7), then eight genogroups were characterized, and now nine (11–16). Given that VP2 is encoded by segment A, these were subsequently termed “A genogroups.” In one proposed system by Islam et al., genogroup A1a contained “classical strains” (with genogroup A1b being vaccine strains), genogroup A2 were antigenically variant strains and divided into sub-lineages A2a, b, and c (13), and genogroup A3 were vv strains. Genogroup A4 were South American strains, genogroup A5 were Mexican recombinant strains, genogroup A6 were Eastern European and Italian strains, and genogroups A7 and A8 were Australian strains, early or variant, respectively (Table 1) (12). Portuguese strains have now been proposed as A9 (14). Two other classification systems were also suggested around the same time: Wang et al*.* proposed that genogroup A8 strains are classical vaccine strains instead of A1b, and all Australian strains are A7 (16), and Gao et al. proposed that attenuated strains are grouped as A9 (11) (Table 1). As the virus is bi-segmented, a region of segment B was also selected for sequencing, and five B genogroups have since been characterized, with B1 termed classic-like, B2 vv-like, B3 early Australian-like, B4 Polish and Tanzanian, and B5 Nigerian (12) (Table 2). IBDV strains are therefore now typed based on their A and B genogroups combined, for example, classical strains are A1B1, US variant strains are A2B1, and vv strains are A3B2 (Table 3).

**TABLE 1:** Genogroups of IBDV segment A

Biological group	Genogroups by the following authors^*a*^			
	Michel & Jackwood	Islam et al.	Wang et al.	Gao et al.
Classical virulent	G1	A1a	A1	A1
Variant	G2	A2	A2	A2
Very virulent	G3	A3	A3	A3
South American	G4	A4	A4	A4
Mexican	G5	A5	A5	A5
Italian	G6	A6	A6	A6
Early Australian	G7	A7	A7	A7
Australian Variant		A8		A8
Attenuated	N/A^*c*^	A1b	A8	A9^*b*^

^*a*^ Table adapted from reference 11, previously published under a CC BY license.

^*b*^ Portuguese strains proposed as A9 to complement systems by Islam et al. and Wang et al. (14).

^*c*^ N/A, not applicable.

**TABLE 2:** Genogroups of IBDV segment B

Biological group	Genogroups by the following authors^*a*^		
	Islam et al.	Wang et al*.*	Gao et al*.*
Classical-like	B1	B1	B1
Very virulent-like	B2	B2	B2
Early Australian-like	B3	B3	B3
Polish and Tanzanian	B4	B4	B4
Nigerian	B5	N/A^*b*^	B5

^*a*^ Table adapted from reference 11, previously published under a CC BY license.

^*b*^ N/A, not applicable.

**TABLE 3:** Examples of common A and B genogroups of IBDV

Biological group	A genogroup	B genogroup
Classical virulent	A1a	B1
Variant	A2	B1
New variant	A2d	B1b
Very virulent	A3	B2
European reassortant	A3	B1

Around 2017, a new variant (nVar) of IBDV emerged in China that was responsible for bursal depletion but had a subclinical presentation, in contrast to the vv strains that were co-circulating at the time (17, 18). Classification of the virus revealed that it had a segment A that was described as A2 lineage d (A2d) and a segment B described as B1b. The A2dB1b virus subsequently spread rapidly and has now been detected in South Korea (19), Japan (20), Egypt (21), and Near East and Persian Gulf countries (22), and has recently been found in Argentina (23). This highlights the need for ongoing global IBDV surveillance; however, the trajectory of nVarIBDV spread is not yet fully understood, and more research is needed to determine the factors that determine the patterns of transmission from country to country. Moreover, since 2023, outbreaks of a mutated vv (mvv) IBDV have been reported across multiple provinces in China, which caused subclinical immunosuppression, associated with antigenic drift mutations in the VP2 capsid, including a D279N mutation that has been associated with decreased virulence (24, 25).

### Detecting IBDV reassortment in the field

IBDV reassortment was initially identified based on incongruence in the phylogenetic trees of segments A and B, and prior to genogrouping, strains were classified as to whether they had a vv or non-vv segment A or B. In 2003, a reassortant strain with a vv segment A and a non-vv segment B (vv A/non-vv B) was first reported in China (26), and in 2006, Le Nouën et al*.* found that 26% of IBDV strains analyzed from 1972 to 2002 in 17 countries contained incongruency in the phylogenetic trees of segments A and B; when one of these was sequenced, it had a vv A/non-vv B (27). Reassortant strains with a vv A/non-vv B have subsequently been reported in China (28–30), Brazil (31), Zambia (32), US (33), India (34–36), Pakistan (37), Algeria (38), Nigeria (39), Europe (22, 40–46), Turkey (47, 48), Iraq (49), and the Near East and Persian Gulf regions (50).

As genogrouping became more common, many of the reassortant viruses with a vv A/non-vv B were found to belong to genogroup A3B1, and this genogroup was found to be widespread in Western Europe. In 2020, they were found to be in Latvia, Germany, Belgium, Denmark, the Netherlands, the Czech Republic, and Sweden (42), with the majority of the viruses likely the result of reassortment between A3B2 vv strains and attenuated A1B1 vaccine strains. Some of these strains also contained mutations Q219L, G254D, D279N, and N280T in the A3 HVR, demonstrating that both antigenic drift and reassortment had occurred, and the A3B1 reassortant viruses were evolving into different clades. In 2023–2024, France, Italy, Portugal, Spain, and the UK were added to the countries where the reassortant A3B1 strains were reported, demonstrating that their geographical range was even more extensive than previously thought (40, 43, 45, 46), and more recently, they have been found in Turkey, India, Iraq, and the Middle East (34, 47–50). Furthermore, in some reports, the A3B1 genogroup was found to be the predominant strain in the field samples, replacing the strains that were previously dominant in the area (46, 47). This increase in the A3B1 reassortant viruses in Europe and surrounding countries represents a major and rapid shift in the epidemiology of IBDV, and it is possible that A3B1 reassortant viruses may continue to spread and become the predominant strain in more countries in the future.

Although less common than the A3B1 reassortants, there have also been reports of reassortant IBDV strains with a vv A/non-vv B that belong to genogroups other than B1. For example, genogroups A3B3 in Bangladesh and China (51, 52), A3B4 in Poland (43), and A3B5 in Nigeria (53), possibly reflecting reassortment between A3B2 vv strains and endemic local strains. In addition, there have been reports of reassortment between IBDV strains with a non-vv A/vv B in China (attenuated A/vv B) (54, 55), USA (A2B2) (33), Turkey (A1B2) (48), and Egypt (A1B2) (56). However, interestingly, the non-vv A/vv B combination appears to be far rarer than the vv A/non-vv B combination, for reasons that remain poorly understood. Moreover, reassortant strains can further reassort with other endemic strains, thus expanding genetic diversity. For example, in China, co-infection with A3B3 strains and endemic A2dB1b strains has been observed (52), as well as the emergence of an A2dB3 reassortant strain (57). Furthermore, there have been reports of reassortment between IBDV strains of different serotypes, for example, a vv A/serotype 2 B virus was identified in Europe (58) and the USA (33, 59), and a serotype 2 A/classical B virus was also identified in the US (33).

The majority of traditional IBDV molecular epidemiological studies relied on Sanger sequencing of a small fragment of the VP2 gene encoded by Segment A that included the HVR, and a small fragment of the VP1 gene encoded by Segment B; hence, the full sequence diversity was often unknown. While this approach was sufficient to genogroup the viruses and assess if inter-genogroup reassortment was occurring, intra-genogroup reassortment was not characterized. For example, in one report from the UK, samples from 50% of farms containing A3B1 reassortant viruses were also found to be co-infected with two different live-attenuated vaccine viruses (A1aB1), and a classical field strain (A1bB1) was also found to be circulating in another sample taken at the same time (46). Therefore, it is possible that there could have been ongoing reassortment between multiple vaccine viruses, classical field strains, and reassortant strains in the field, but as they all possessed a segment B1 and the whole segment was not sequenced, this remains unknown.

In recent years, whole genome sequencing (WGS) approaches have been employed to the study of IBDV and have identified intra-genogroup reassortment as a result. For example, Wang et al*.* (60) identified a reassortant virus in China that contained a segment A from a classical field strain and a segment B from an attenuated vaccine strain (60), and Feng et al*.* identified the opposite—a strain with a segment A from a vaccine strain and a segment B from a classical strain (61). It is anticipated that the application of next-generation sequencing (NGS) technology to the study of IBDV will lead to more discoveries of intra-genogroup reassortment in the future, but it will be necessary to utilize long-read sequencing approaches.

In addition to reassortment, *Birnaviridae* can undergo homologous recombination in co-infected cells, where the polymerase replicates one genome segment that contains part of the sequence from one strain and part from another strain (62–64). In addition, evidence of both recombination and reassortment has been observed in some isolates. For example, the segment A of the reassortant viruses identified in China by Wang and Feng et al. displayed potential recombination events (60, 61). Moreover, in 2025, Paramasivam et al*.* employed six bioinformatics algorithms to identify recombination events and discovered that out of eight A3B1 reassortant isolates in India, four recombination events were predicted to have taken place in Segment A, and nine in Segment B, with each individual strain having 1–2 recombination events (34). It is anticipated that the increasing application of NGS technologies and bioinformatics tools to IBDV research will lead to an increase in the discovery of recombination events in the future, but it will be also necessary to utilize long-read sequencing technologies.

### Potential reservoirs of IBDV gene segments

The emergence of the first vv IBDV strains in the 1980s was thought to be the result of a reassortment event between IBDV in domestic poultry and an as yet unknown source of a divergent segment B (65, 66). Although the donor of the segment B was never identified, it has been proposed that wild birds could harbor strains of avibirnaviruses and act as reservoirs of gene segments. Surveillance for IBDV in wild birds has been limited; however, a meta-analysis and systematic review of 33 studies placed the pooled seroprevalence at 6% (95% confidence intervals 3%–9%) in wild bird populations (67). Six studies attempted to detect IBDV by reverse transcription polymerase chain reaction (RT-PCR) from tissue samples or cloacal swabs, and four found viral antigen in Anseriformes, Passeriformes, Columbiformes, and Pelecaniformes in addition to Galliformes with a reported prevalence of 3.8% (68), 4.7% (69), 9.1% (70), and 10.7% (71). Moreover, the sequences of these viruses had 99.26%–99.85% identity with the sequences of known IBDV strains isolated from poultry belonging to genogroups A3 and A1, suggesting that wild birds could play a role in the ecology of IBDV, although it is unclear whether these represented spillover events from poultry into wild birds. Moreover, one sequence had 99.48% identity with a live-attenuated IBDV vaccine strain, suggesting that wild birds could be exposed to live vaccine strains, for example, if they encounter poultry facilities. Once in wild bird populations, differing selection pressures could lead the IBDV viruses to evolve differently, which could potentially complicate control efforts should they spill back into poultry or reassort with poultry strains.

Moreover, in 2022, Hill et al*.* used a metagenomics approach to characterize the viral and bacterial populations present in feces collected from a healthy population of wild mute swans (*Cygnus olor*), and as part of this process, they identified a novel avibirnavirus (72). The full-length genome sequence was obtained, and a nucleotide BLAST search revealed that segment A only had a maximum of 85.61% identity with known IBDV serotype 2 viruses and 85.02% identity with known IBDV serotype 1 viruses. This suggests that the avibirnavirus was a novel wild-bird birnavirus that potentially belongs to another serotype. It is therefore possible that wild birds can harbor avibirnavirus segments that have not previously been detected in domestic poultry, and the potential exists for reassortment with domestic poultry strains. If such an event were to occur, this could lead to the emergence of novel strains in the future that could be a threat to the poultry industry.

### Consequences of IBDV reassortment

It is known that both segments A and B contribute to the virulence of IBDV (73). Therefore, reassortment events can alter the clinical presentation of infection and the degree of pathology observed in the BF. For example, the vv A/non-vv B reassortant viruses belonging to genogroup A3B1 isolated in Europe typically cause no significant mortality or clinical signs, but still lead to marked bursal atrophy following experimental inoculation of specific pathogen-free (SPF) chickens (41–43, 49). This is in contrast to the A3B2 vv viruses that lead to high morbidity and mortality percentages. Therefore, the substitution of the polymerase from B2 to B1 could contribute to viral attenuation, although it should be noted that some of the antigenic drift mutations that have arisen in the VP2 capsid protein of the A3B1 reassortant viruses can also be attenuating (25). As A3B1 reassortant strains are becoming more prevalent in Europe, it is possible that poultry producers and veterinarians in this part of the world may start to notice less severe clinical signs and mortality in the field associated with vv IBDV, and instead, more asymptomatic/subclinical infections that are nevertheless highly immunosuppressive. This could complicate control efforts, as it is likely that birds will initially present with a secondary infection that leads professionals to suspect an underlying immunosuppression. Similarly, in the US, inter-serotypic reassortment producing a virus with a vv A/Serotype 2 B also led to no mortality following experimental challenge of chickens, but nevertheless caused bursal atrophy (33).

Reassortant viruses with a non-vv A/vv B can also show clinical presentations that are intermediate between vv and non-vv viruses. For example, Wei et al*.* described how a non-vv A/vv B reassortant virus isolated in China caused 20% mortality in SPF chickens, compared to a vv strain which caused 40% mortality, and a non-vv strain that caused no mortality (74), and Mosad et al*.* describe how an A1B2 reassortment virus isolated in Egypt created bursal lesions similar to the vv strain, but less severe (56). Furthermore, the A2dB3 reassortant strain that occurred following reassortment of A3B3 strains with A2dB1b viruses led to 10% mortality in SPF chickens, in contrast to 0% mortality with the A2dB1b virus (57).

In the US, Jackwood et al*.* discovered that a reassortant with a vv A/non-vvB was less pathogenic than the vv IBDV strain (rB) but more pathogenic than the non-vv classical strain (STC), consistent with the reports from Europe. Similarly, reassortant viruses with a vv A/Serotype 2 B showed 20%–70% mortality, which was intermediate compared to the vv strain which caused 100% mortality, and the serotype 2 virus which caused 0% mortality (33). Moreover, in the same study, a reassortant virus with a subclinical A/vv B caused more bursal pathology than the subclinical virus alone (Del-E), consistent with the reports from China concerning the pathogenicity of non-vv A/vv B strains. This study also confirmed the 1980s observations that vv viruses could break through maternal antibodies conferred by traditional vaccines, and extended these observations by demonstrating that reassortant viruses with either a vv A or vv B segment could also break through maternal immunity induced by classical or subclinical vaccines. The authors concluded that broiler breeder vaccination programs aimed to protect against “classical” strains might not adequately protect against reassortant strains containing vv segments (33).

Taken together, IBDV reassortment was first reported in the early 2000s, but recently, there has been a rapid increase in A3B1 reassortant strains in Europe and elsewhere that is responsible for subclinical infection that could complicate control efforts. We anticipate that the increasing application of WGS approaches, NGS technology, and bioinformatic pipelines to the study of IBDV will further identify intra-genogroup reassortment and concurrent reassortment and recombination events that will enable us to better define the genomic sequence diversity of IBDV, both in poultry and in wild bird populations.

## IPNV REASSORTMENT

### Introduction to IPNV

IPNV primarily affects salmonid species, such as Atlantic salmon (*Salmo salar*) and rainbow trout (*Oncorhynchus mykiss*), particularly during first feeding and in the early weeks following transfer to seawater. IPNV was first characterized in 1960 (75), and the virus is thought to enter the body via the oral cavity and gills and potentially also through breaches in the skin, after which it spreads systemically through the circulatory system. The virus can subsequently be detected in the kidney, spleen, pancreas, liver, heart, brain, as well as the skin, intestine, and reproductive cells, but the main target organs are the pancreas and liver, where infection leads to extensive necrosis of pancreatic acinar cells and necrotic lesions within hepatic tissue. Clinically affected fish typically display darkened skin pigmentation and swim abnormally in a corkscrew or spiral motion, and necropsy findings commonly include abdominal distension, vascular congestion, petechial hemorrhages in the pyloric caeca, and a pale or friable liver (76, 77). The virulence of IPNV is highly variable, with mortality rates ranging from 10% to 90%, depending on the viral strain, host species and genetics, infectious dose, environmental conditions, stressors associated with husbandry practices, and the age of the fish at the time of exposure. Transmission of IPNV occurs predominantly through horizontal spread, although the virus has been detected in gonadal fluids and has been shown to bind spermatozoa, meaning egg-associated infection is a risk. Fish that survive typically develop protective immunity and show normal growth when moved to seawater; however, some individuals can become chronically infected, becoming lifelong carriers and may either show no clinical signs or reduced growth performance but can nevertheless intermittently shed the virus, thereby serving as persistent reservoirs for transmission (76, 77).

The virus is thought to have a widespread global distribution, and most major salmon-producing countries are affected; however, the reports of IPNV are not evenly distributed geographically (76). Typically, fish producers mitigate the impact of the virus through vaccination, routine surveillance, and biosecurity measures at aquaculture facilities. Moreover, since 2009, selective breeding programs targeting an IPN-associated quantitative trait locus (QTL) that was subsequently mapped to the E-cadherin gene (*cdh1*) (78) have led to a reduction in IPN Norway, but in recent years, viral variants that appear to evade this protection have been identified, raising concerns about the long-term durability of genetic resistance strategies (79).

IPNV strains were historically named based on the place of isolation or scientists involved, for example, West Buxton (WB), Jasper, Abild (Ab), Tellina (Te), Canada (Can)1, Can2, Can3, Spjarup (Sp), and Hecht (He). The strains were then classified into two broad serogroups (A and B), which together encompass 10 serotypes: A1–A9 and B1. The serological classification partially mirrored the geographic distributions of the strains, with serotype A1 predominating in the US and including strain WB, serotypes A2–A5 frequently including European and Chilean isolates for example Sp, Ab, He, and Te, and serotypes A6–A9 largely originating from Canada, for example, Can1 (A6), Can2 (A7), Can3 (A8), and Jasper (A9). As with IBDV, molecular approaches refined IPNV classification, and initially six, then seven genogroups were identified based on segment A (I–VII), and three major genotypes were described based on segment B (Groups 1, 2, and MABV). For example, genogroup I contains strains WB and Jasper, genogroup II corresponds to strain Ab, genogroup III contains strains Te and Can1, genogroup IV contains Can2 and Can 3, genogroup V contains European and Chilean Sp isolates, and genogroup VI contains He. Genogroup VII is proposed to contain Japanese aquatic birnaviruses (79) (Table 4).

**TABLE 4:** Examples of common A and B groups of IPNV

Strain	Common name	A serotype	A genogroup	Putative B group
WB	West Buxton	A1	I	1
Sp	Spjarup	A2	V	2
Ab	Abild	A3	II	2
He	Hecht	A4	VI	2
Te	Tellina	A5	III	2
Can1	Canada 1	A6	III	2
Can2	Canada 2	A7	IV	2
Can3	Canada 3	A8	IV	2
Jasper	Jasper	A9	I	1

### Detecting IPNV reassortment in the field

IPNV reassortants were first reported in 2009 in natural fish populations from the Flemish Cap fishery at Newfoundland following WGS (80). While most isolates were strain WB, and one was strain Ab, a reassortant was observed with a WB segment A and an Ab segment B (WB/Ab). Reassortant viruses with a WB segment A and either an Ab or a Jasper segment B were subsequently found in wild fish around the Gulf of Cadiz (81), and reassortant strains with a segment A from a genogroup III virus and a segment B from a genogroup II virus were detected in Scotland (82). Panzarin et al*.* also described the detection of a putative reassortant IPNV with a genogroup V segment A and Group 2 segment B (83). However, reports of IPNV reassortment are rarer than IBDV reassortment. For example, Panzarin et al*.* analyzed the samples from 1978 to 2017 and only found evidence of one reassortment event, but the underlying reasons for this remain poorly understood.

### Consequences of IPNV reassortment

Romero-Brey et al*.* compared the phenotype of IPNV strains *in vitro* and found that an Ab isolate (6B1-A) replicated to lower titers than the WB strains in CHSE-214 cells, and the WB/Ab reassortant (isolate 20G1-B) had intermediate replication kinetics, initially replicating less than the WB strains, but eventually reaching the same peak titer (80). Furthermore, following *in vivo* challenge of sole, strain Ab caused 30% mortality, and WB caused 0% mortality, whereas the WB/Ab reassortant strain caused 3% mortality. In salmon, Ab caused 100% mortality, WB caused 0% mortality, and the WB/Ab virus caused 18% mortality (84). These data suggest that as with IBDV, reassortant IPNV strains can have a phenotype intermediate to that of the parental strains. However, this was not always the case, as the Sp strain caused 16% mortality in salmon, but the WB/Sp reassortant strain caused 0% mortality.

## MOLECULAR BASIS OF REASSORTMENT, DRIVERS, AND CONSTRAINTS

For reassortment to occur, one cell must be infected with two or more birnaviruses, and their genome segments must be able to co-mix and become packaged into newly synthesized virions. Therefore, to better understand the molecular basis underpinning the phenomenon, it is necessary to better understand the cell biology of birnavirus infection.

### Birnavirus replication cycle

Compared with other dsRNA families, for example, the *Reoviridae*, less is known regarding the birnavirus replication cycle, and this is an active area of research. The cycle begins with attachment of the viral particle to the host cell and the uptake of the virion into an endosome. A decrease in pH and calcium ion concentration in the endosome promotes disassembly of the single-layered capsid, releasing a small amphipathic peptide (pep46) that promotes pore formation in the endosomal membrane (85), through which the viral genome segments enter the cytoplasm (86). Each genome segment exists as a viral ribonucleoprotein (vRNP) complex where the dsRNA is coated by VP3 and covalently linked to the VP1 protein at the 5′ end, which acts as a 5′ cap. The vRNPs then associate with the cytoplasmic leaflet of endosomal membranes (86, 87) and seed the formation of biomolecular condensates known as “virus factories” (VFs) that form through liquid-liquid phase separation (LLPS) and grow due to both the synthesis and accumulation of viral components, and the coalescence and fusion of multiple VFs (5, 88–90). The VFs are thought to be the site of viral genome replication, which is driven by the VP1 polymerase, and VP1 interacts with VP3 for optimal function (91–93). Viral particles subsequently form, and it is thought that upon the death of the host cell, virions are released by cell lysis; however, IBDV-infected cells can also shed virus in a non-lytic manner, a process that has been reported to be mediated by protein VP5 (94).

### Molecular basis of birnavirus reassortment

Previously, it has been shown that when one cell is experimentally coinfected with two IBDV lab-adapted strains expressing different fluorescent markers tagged to VP1, initially separate discrete VFs appear in the cytoplasm from each parental strain that eventually coalesce over time and mix together (89). However, it remains unknown whether VF coalescence is required for reassortment to occur. Related to this, it is also unknown whether newly synthesized viral RNA remains in the VF or whether it is able to leave the factory and enter the cytoplasm, for example, for translation. If it remains in the VF, then it is conceivable that VF coalescence is required for reassortment to occur, whereas if it is capable of leaving the factory and then being recruited back, then it may be possible for reassortment to proceed in the absence of VF coalescence.

Moreover, within the VF, the mechanism of genome packaging and virion assembly is only beginning to be understood in the *Birnaviridae* family, compared to the more well-studied *Reoviridae* family. Reoviruses have 9–12 genome segments and have evolved highly selective packaging systems where the positive-sense RNA component of each segment is packaged into newly assembled virus core particles. However, it is currently thought that genome incorporation into a birnavirus particle occurs during capsid assembly, where multiple VP3 proteins combine to form an irregular shell that binds VP2 capsid proteins to form a “procapsid,” while also binding the dsRNA genome segments and VP1 polymerases, thus providing a “scaffolding” function, bridging the genome and the capsid proteins to build the virus particles (95, 96). Moreover, birnaviruses have been reported to be polyploid, and virus particles have been identified with 1, 2, 3, or 4 gene segments inside (96–98). This method of encapsidation and polyploidy means birnaviruses are thought to undergo so-called semi-random assortment, meaning that while one infectious unit requires one segment A and one segment B (AB), some virus particles may contain A, B, AA, BB, AAB, BBA, AABB, ABBB, or BAAA, and any particle containing at least one A or one B would be infectious. The molecular basis underpinning polyploidy remains to be determined; however, as the dsRNA genome is packaged as vRNP complexes with VP1 and VP3, it is possible that differences in how efficiently each segment binds VP1 and VP3 could bias whether A and B are co-packaged, whether extra copies are included, or whether incomplete particles form. Furthermore, viral variants that differ in VP1-RNA linkage efficiency or vRNP stability might alter the probability that a particle contains A only, B only, A + B, or multiple copies.

One consequence of polyploidy is that in coinfected cells, there could be virus particles that contain a combination of gene segments from both parental strains. Moreover, when a new birnavirus isolate is sequenced, it is important to use long-read NGS technology that can discern if multiple As or Bs are present in the sample, and previous reports that relied on Sanger sequencing alone may have only reported the most dominant sequence, or the consensus sequence present. Furthermore, if a bursal sample was sequenced and multiple IBDV segments A and B were found, it was traditionally labeled as a co-infected sample with the presumption that the bursa was infected with multiple IBDV strains. However, it is difficult to rule out whether the sample was truly co-infected with multiple viruses at the same time or whether it was infected with one reassortant virus that contained multiple As and Bs from different parental strains. One way to dissect this phenomenon would be to plaque-purify the virus isolates prior to sequencing the genome; however, this requires the virus to replicate within an adherent cell line, and as the field strains of IBDV have a preferred tropism for B lymphocytes, this is not always possible.

### Drivers and constraints to reassortment

Key questions remain regarding reassortment in the *Birnaviridae* family. For example, what are the constraints on reassortment? Inter-generic reassortment has not been observed, for example, between IPNV and IBDV strains, but whether this is because of a molecular incompatibility or because the two viruses occupy different ecological niches so that there is never an opportunity to co-infect an organism and reassort remains to be determined. Additionally, reassortment between IPNV strains seems to be far rarer than between IBDV strains, for reasons that remain poorly understood. While it could be in an aquatic environment the viral pressure/burden may be less than in poultry units, meaning that co-infection is inherently less likely, there could also be a molecular basis for the observation. To investigate this further, Lago et al*.* co-infected immortalized cells with different IPNV strains and quantified the percentage of reassortment that resulted. When WB and Cu1 IPNV strains were mixed together, the majority of viruses recovered were reassortants, demonstrating that reassortment is not inherently constrained in IPNV populations. However, when Ab and WB were mixed together or when Ab and Sp were mixed together, very little reassortment resulted. There were also cellular factors involved, as different patterns of reassortment were observed when the experiment was repeated in different cell lines. The authors conclude that there are biases involved in IPNV reassortment, and they discussed several possibilities for the observation: first, if the transcription and/or replication efficiency of one of the genomes in the co-infection is higher than the other, it would be over-represented in the viral population. Second, different mRNAs from the different viral strains may be translated at different levels (possibly related to the ability of viral molecules to interact with cellular translation initiation factors). Third, there may be an association of certain segments with each other (84). This could be because of physical interactions between the vRNP complexes of the different segments. For example, a segment from one strain may not form an optimal vRNP complex with the VP1 or VP3 from a different strain. Therefore, a reassortant virus carrying a segment A from one strain and B from another may be selected for or against depending on whether the heterologous VP1 efficiently recognizes, replicates, caps, or packages the A-segment RNA. Moreover, reassortant viruses with poorly matched A/B combinations may fail to complete their replication cycle after cellular entry because the VP1 must replicate and transcribe the heterologous segment. Finally, VP1 and VP3 interact with one another, and this interaction increases VP1 polymerase activity. As these proteins are encoded by different segments, there may be strain-dependent differences in the compatability of this interaction that constrains reassortment.

These same factors could be at play in IBDV. There are over 27 reports describing reassortants with a vv A/non-vv B combination, but to our knowledge, only five reports have described reassortants with a non-vv A/vv B; however, the reason for this bias remains unknown. It is possible that the combination of non-vv A/vv B leads to a fitness cost in the progeny compared to parental strains, but this remains to be determined. The converse might also be true: that the combination of a vv A/non-vv B might be more fit than the parental strains. The fact that A3B1 strains are increasingly being found in Europe implies that they could be more biologically fit than the parental vv A3B2 or classical and vaccine A1B1 strains; however, this remains to be determined.

One other constraint on the ability of reassortment to proceed is the window period over which cells can be co-infected. Whether a cell must be co-infected with two birnaviruses at the same time to establish reassortment or whether they can be infected with one birnavirus and then super-infected with another remains unknown. The phenomenon of super-infection exclusion (SIE) exists in some viruses, where infection with the first virus excludes infection with the second. In IBDV, there is one report of a vaccine virus providing partial protection against a field strain when given 24 h prior to challenge (99). As this is insufficient time for protection to be mediated by adaptive immunity, it is possible that the mechanism was based on innate immunity or SIE; however, this remains an understudied area in the *Birnaviridae,* and defining the window period over which co-infection can occur is important, as the narrower the window, the less likely reassortment is to proceed.

Modeling the factors that affect reassortment is necessary to better understand how novel reassortant strains emerge. To do this, it is first necessary to quantify the baseline reassortment efficiency in the absence of confounding factors, between strains that are similar to each other, before then comparing reassortment efficiency between heterologous strains, or under different conditions. However, most reassortment detection methods rely on parental viruses being sufficiently different from each other to distinguish between the strains, which then introduces complications such as fitness differences among the progeny due to segment mismatch. What is needed is a method to quantify unbiased reassortment, in the absence of segment mismatch. This has been achieved for influenza viruses through the insertion of a synonymous point mutation into every genome segment. As the mutations are synonymous, viral fitness remains the same, but it is possible to distinguish mutant from wild-type (wt) segments by RT-qPCR and high-resolution melt-curve analysis (100). This method was used to determine the baseline frequency of influenza A virus reassortment, and the impact of infectious dose and timing *in vitro*, and the method was subsequently applied *in vivo* to determine the intra-host dynamics of reassortment in a guinea pig model (101). By systematically varying individual parameters, the impact of a wide range of factors on the frequency of influenza virus reassortment was modeled, including reassortment of heterologous strains, the effect of anatomical compartmentalization on reassortment, the transmission of reassortant strains, reassortment in different species, and the effect of packaging signals on reassortment (102–109). In 2020, the method was expanded to quantify reassortment in the dsRNA virus family *Reoviridiae* (100). Given that it is not possible to accurately make predictions regarding the biology of the *Birnaviridae* based on data from the *Reoviridae,* the factors affecting *Birnaviridae* reassortment are likely to be different from those affecting the *Reoviridae*, and a method to quantify *Birnaviridae* reassortment would allow us to model the drivers and constraints to reassortment, the conditions that are most favorable for reassortment, and the circumstances that give rise to novel strains in nature.

## GAPS IN OUR KNOWLEDGE

One major gap in our understanding of reassortment in the *Birnaviridae* family is the characterization of reassortment in birnaviruses other than the *Avibirnavirus o*r *Aquabirnavirus* genera. To our knowledge, there are no reports of reassortment in the *Blosnavirus*, *Telnavirus*, *Ronavirus, Dronavirus,* or *Entomobirnavirus* genera, presumably because less surveillance has been conducted. Additionally, more surveillance is also needed in fish to fully understand the extent of reassortment in the *Aquabirnavirus* genus, and more surveillance is needed in wild birds to fully determine the diversity of *Avibirnavirus* segments that could potentially reassort with common domestic poultry strains. In addition, although inter-genogroup reassortment has been characterized in IBDV and IPNV populations, our understanding of the prevalence of intra-genogroup reassortment is lacking, and WGS approaches using long-read sequencing technologies are needed to answer this important question. Moreover, it is also important to use NGS approaches to discern if multiple segment As or Bs are present in a sample when a new birnavirus isolate is sequenced, although without the ability to plaque-purify field strains of IBDV, it will be difficult to determine if the presence of multiple As and Bs represents co-infection or reassortment.

Regarding the molecular and cellular basis for reassortment, we do not yet know whether VF coalescence is required for the phenomenon to occur or whether viral RNA can leave a VF. Furthermore, the molecular drivers and constraints to birnavirus reassortment remain poorly characterized, for example, whether there are certain combinations of IBDV or IPNV segments that are either favored or disfavored and the molecular basis underpinning this, or an understanding of whether SIE occurs in the *Birnaviridae* family. Developing methods to quantify reassortment frequency in the absence of fitness differences will enable us to investigate these fundamental aspects of the biology of the viruses in the future.

## CONCLUDING REMARKS

Investigations into reassortment in the *Birnaviridae* began in the early 2000s, initially by comparing incongruencies in the phylogenetic trees constructed from Sanger sequences of small regions of the viral genome. Since then, molecular genogrouping has allowed us to better annotate when reassortment has occurred, and NGS approaches as well as pipelines for WGS of birnaviruses have opened up new avenues of research into intra-genogroup reassortment and the co-existence of reassortment and recombination events. Continued technological advances and surveillance will allow us to fully understand the sequence diversity in the *Birnaviridae* in the future, but new studies are also needed to better understand the fundamental molecular basis underpinning the phenomenon. Regarding IBDV specifically, the increase in reassortant viruses in Europe represents a recent major shift in the epidemiology of the virus, and it is possible that their geographic range could continue to expand in the future, leading to atypical clinical presentations, subclinical immunosuppression, and potentially hindering control efforts. Finally, the fact that the majority of these strains could be the result of reassortment between circulating field strains and attenuated/vaccine strains means that we may have inadvertently contributed to the increasing complexity of IBDV epidemiology through decades of live vaccine use.
